# Halotolerant Rhizobacteria from Phragmites Communis: A Controlled Proof-of-Concept for Crop Improvement in Degraded Sandy Soils

**DOI:** 10.3390/microorganisms14051120

**Published:** 2026-05-14

**Authors:** Kadir Sinan Arslan, Meriam Bouri, Aissa Bakelli, Fikrettin Şahin

**Affiliations:** 1 Department of Genetics and Bioengineering, Faculty of Engineering, Yeditepe University, Istanbul 34755, Türkiye; 2 Department of Biology, Faculty of Natural and Life Sciences and Earth Sciences, University of Ghardaia, Ghardaia 47000, Algeria

**Keywords:** halotolerant bacteria, PGPR, microbial diversity, Saharan rhizosphere, *Triticum aestivum*, *Capsicum annuum*, micronutrient availability

## Abstract

Halotolerant plant growth-promoting rhizobacteria (PGPR) represent a promising strategy for enhancing crop productivity in degraded soils. This study evaluated 51 bacterial strains isolated from the rhizosphere of the Saharan halophyte *Phragmites communis* L. for their capacity to improve the performance of wheat (*Triticum aestivum* L.) and pepper (*Capsicum annuum* L.) under nutrient-deficient sandy soil conditions. The selection of halotolerant isolates was based on their potential for cross-tolerance, assuming that their adaptive mechanisms against salinity could also mitigate the osmotic and nutritional constraints inherent to nutrient-poor sandy substrates. Two strains, XE-15 and XR-18, were selected based on in vitro screening and tentatively assigned to the genera *Pseudomonas* and *Bacillus*, respectively, using 16S rRNA sequencing and multilocus sequence analysis (MLSA). Greenhouse experiments demonstrated that bacterial inoculation significantly increased plant biomass (up to ~2-fold compared to control) and enhanced pepper fruit yield (0.68 g vs. 0.20 g in control). XR-18 notably increased Fe (up to 198.65 mg kg^−1^) and P (7.98 mg kg^−1^) accumulation in wheat, while XE-15 exhibited substantial concentrations of nitrogen (1.08%) and magnesium (4.11 mg kg^−1^) and zinc (102.3 mg kg^−1^). Soil properties were also improved, including increased water-holding capacity (~30%) and enhanced micronutrient availability. Zinc showed the most pronounced strain-specific response, increasing by 84% under XE-15 and by more than 160% under XR-18. However, taxonomic resolution remains tentative in the absence of genome-level analyses, and mechanistic insights are primarily inferred from in vitro traits. The simplified greenhouse system further limits ecological interpretation. These findings highlight the potential of halotolerant PGPR in degraded soils while emphasizing the need for genomic validation, mechanistic studies, and field-scale evaluation.

## 1. Introduction

Soil fertility and precipitation are among the principal determinants of global food security under conditions of ongoing climate change and rapid population growth. Increasingly frequent extreme weather events (including floods, strong winds, temperature extremes, drought, and frost), which can trigger environmental disturbances such as landslides, are severely disrupting soil functions and accelerating degradation processes. Meanwhile, the global population continues to grow by approximately 73 million people per year [1]. In combination with intensive agricultural practices, deforestation, urban expansion, and industrial activities, this demographic pressure places unprecedented stress on soil resources. Consequently, demands on agricultural land are expected to intensify substantially in the coming decades.

Soil degradation is expressed through a wide range of interconnected biological, chemical, physical, and ecological processes, including erosion, salinization, acidification, desertification, nutrient depletion, compaction, and heavy metal contamination [2,3,4]. In response, numerous strategies aligned with the Sustainable Development Goals have been implemented at various governance levels to prevent and remediate agricultural soil degradation. At the field scale, recommended practices include conservation tillage, crop rotation, strip cropping, efficient irrigation management, reduced application of chemical fertilizers and pesticides, and terrace farming on sloping land, all of which aim to preserve soil health and fertility [5,6].

In parallel, microbe-based approaches have emerged as key components of sustainable land management and have attracted growing attention from both the scientific community and policymakers. Since soil fertility is governed by the interaction of its physical, chemical, and biological properties, maintaining and enhancing microbial diversity involved in nutrient cycling is widely recognized as essential for ensuring adequate nutrient availability and meeting plant nutritional requirements for optimal productivity [7,8]. More recently, the deliberate management of plant-associated microbiomes (particularly rhizospheric microbial communities) has been identified as one of the most promising strategies for promoting plant growth and resilience in sustainable agriculture systems [9]. Rhizospheric microbiomes play fundamental roles in soil bioremediation [10], climate change mitigation [11], and plant nutrient acquisition [12]. Soil microorganisms contribute to the cycling of essential nutrients such as nitrogen, phosphorus, and carbon through the mineralization of soil organic matter and the development of stable soil structure [13].

Among these microorganisms, plant growth-promoting rhizobacteria (PGPR) enhance soil fertility and crop productivity via multiple direct and indirect mechanisms, including biological nitrogen fixation; solubilization and mineralization of phosphorus, zinc, and other nutrients; production of phytohormones and siderophores; synthesis of antimicrobial and enzymatic compounds that improve tolerance to abiotic and biotic stresses; and the ISR (Induction of Systemic Resistance) in plants [14]. For example, nitrogen-fixing rhizobacteria such as *Azospirillum* spp., *Azotobacter* spp., *Bacillus* spp., and *Enterobacter* spp. are capable of converting atmospheric nitrogen into plant-assimilable forms, thereby increasing nitrogen availability in the soil [15]. In addition, certain bacterial and fungal taxa produce extracellular polymeric substances, including exopolysaccharides and mucilage, which promote soil aggregation by binding soil particles into stable microaggregates and enhancing soil structural integrity [16]. Moreover, microbial strains isolated from saline and arid environments have demonstrated considerable potential in alleviating salt stress [17,18] and drought stress [19].

Within this context, the present study aimed to isolate and identify potential PGPR from halophytic plants and saline–alkaline soils of the Sahara. The performance of selected bacterial strains, XE-15 and XR-18, was evaluated in pot experiments using wheat (*Triticum aestivum* L.) and pepper (*Capsicum annuum* L.) cultivated in degraded sandy soils without the application of chemical fertilizers. Plant growth parameters and post-cultivation soil physicochemical properties were systematically assessed to elucidate the effects of bacterial inoculation on crop performance and soil functional properties.

## 2. Materials and Methods

### 2.1. Isolation of Bacterial Strains

Bacterial strains were isolated from the rhizosphere of *Phragmites communis* L. growing in saline–alkali soils of the Ghardaia Sahara, Southern Algeria. Isolation was conducted on Nutrient Agar (NA) medium (Merck, Darmstadt, Germany). Plant roots were washed twice with sterile distilled water (SDW) to remove adhering particles. Subsequently, 10 g of rhizospheric soil was suspended in 90 mL of SDW and agitated at 150 rpm for 30 min. Serial dilutions were spread onto NA plates and incubated at 25 °C for 72 h. Pure cultures were initially characterized by Gram staining and preserved in 30% (*v*/*v*) glycerol at −20 °C for long-term storage.

### 2.2. Screening for Plant Growth-Promoting (PGP) Traits

Nitrogen fixation activity was assessed using semisolid NFb medium as described by Döbereiner [20]. Phosphate solubilization was evaluated on Pikovskaya medium containing 5 g L^−1^ tricalcium phosphate (Ca_3_(PO_4_)_2_) [21]. Inorganic potassium solubilization was identified using Aleksandrov agar medium after incubation at 25 ± 2 °C for 48 h.

Indole-3-acetic acid (IAA) production was quantified according to Patten and Glick [22]. Strains were cultivated in Nutrient Broth (NB) supplemented with L-tryptophan (500 μg mL^−1^) and incubated for four days at 30 ± 2 °C. Cell-free supernatants were mixed with Salkowski’s reagent (containing 10 mM H_3_PO_4_) and incubated in the dark for 25 min; pink coloration indicated IAA production. Siderophore production was detected on chrome azurol S (CAS) agar [23]. Orange halos surrounding colonies after 48–72 h at 25 °C confirmed iron-chelating activity. Zinc solubilization was assessed on Tris-minimal agar supplemented with 0.1% (*w*/*v*) ZnO [24].

### 2.3. Enzymatic Activity Assays

Amylase production was screened on starch agar using iodine solution to visualize clear zones [25]. Protease activity was determined on skim milk agar [26]. Lipase activity was assessed on media containing tributyrin or Tween 80 [27]. Cellulase activity was examined on carboxymethyl cellulose (CMC) agar, stained with Congo red [28].

### 2.4. Phylogenetic Characterization and Multilocus Sequence Analysis (MLSA)

Phylogenetic characterization of bacterial strains XE-15 and XR-18 was initially conducted through 16S rRNA gene sequencing. To achieve higher taxonomic resolution, a multilocus sequence analysis (MLSA) approach was subsequently implemented using genus-specific housekeeping genes. For *Pseudomonas* strains, the *rpoB* and *gyrB* loci were analyzed according to methodologies described by Ait Tayeb et al. [29] and Yamamoto et al. [30]. For *Bacillus* strains, MLSA was performed using the *rpoB*, *groEL*, *gyrA*, and *purH* genes following the protocol established by Ben Gharsa et al. [31].

Strains were cultured overnight in Nutrient Broth (NB) at 28 C with agitation at 120 rpm. Genomic DNA was extracted using a commercial DNA extraction kit (Invitrogen, Thermo Fisher Scientific, Waltham, MA, USA) following the manufacturer’s instructions. PCR amplifications were performed using the Taq PCR Kit (Thermo Fisher Scientific, Waltham, MA, USA) with the following thermal cycling profile: an initial denaturation at 95 °C for 5 min; 35 cycles of denaturation at 95 °C for 30 s, annealing at 55 °C for 30 s, and extension at 72 °C for 2 min; followed by a final extension step at 72 °C for 5 min. The resulting amplicons were purified and subjected to bidirectional Sanger sequencing by Triogen (Triogen, Istanbul, Türkiye).

Raw sequence chromatograms were visually inspected, and high-quality consensus sequences were generated. Multiple sequence alignments were performed using the MUSCLE (v3.8) algorithm implemented in SeaView v4, followed by manual trimming to ensure sequence comparability across datasets. Individual gene trees were initially reconstructed using the Neighbor-Joining (BioNJ) method, and branch support was evaluated through 1000 bootstrap replicates. To assess the robustness of phylogenetic relationships, maximum likelihood (ML) analyses were additionally performed using PhyML (v3.0) under the General Time Reversible (GTR) nucleotide substitution model with 1000 bootstrap replicates.

Reference sequences were retrieved from published datasets representing the *Bacillus subtilis* species complex (taxid: 653685) and the genus *Pseudomonas* (taxid: 286). Orthologous sequences from *Bacillus subtilis* subsp. *spizizenii* (taxid: 96241) and *Stutzerimonas kunmingensis* (taxid: 1211807) served as outgroups for *Bacillus* and *Pseudomonas* analyses, respectively. Prior to concatenation, individual gene trees were examined for topological congruence. Only loci displaying consistent phylogenetic signals were concatenated in the following order: *rpoB*, *gyrB* for *Pseudomonas* and *rpoB*, *groEL*, *gyrA*, *purH* for *Bacillus*.

### 2.5. Seed Germination and In Vivo Greenhouse Assays

Seeds of wheat (*Triticum aestivum* L.) and red pepper (*Capsicum annuum* L.) (Yüksel Tohum, Antalya, Turkey) were surface-sterilized (70% ethanol for 1 min, 2% NaOCl for 3 min). Bacterial cultures were adjusted to 1 × 10^8^ CFU mL^−1^. Germination was assessed after 10 days using ImageJ v1.52s.

Greenhouse experiments utilized 1500 g of sterile, nutrient-deficient sandy soil–perlite–peat substrate (3:1:1 *w*/*w*; pH 6.5–7.2). Soil was inoculated to reach 1 × 10^8^ CFU g^−1^ soil. *Bacillus megaterium* M3 [32] served as a positive control. Plants were grown under a 14 h light (30 °C)/10 h dark (24 °C) photoperiod for 17 weeks without supplemental fertilization.

### 2.6. Soil Characterization

The marginal sandy soil used in this study was collected from the Samandıra district (Sancaktepe, Istanbul, Türkiye). The samples were ground and sieved through a 2 mm mesh before physical and chemical assessment. Particle size distribution was determined using the Robinson pipette method as described by Tolno et al. [33]. Soil pH and electrical conductivity (EC) were measured in a 1:3 (*w*/*w*) soil-to-water suspension using a calibrated pH meter and an EC meter (Mettler Toledo, Columbus, OH, USA), respectively.

### 2.7. Plant Growth Parameters Assessment

After 17 weeks of cultivation, plants were harvested to evaluate root and shoot lengths, as well as fresh and dry biomass. Dry weight was determined by oven-drying the plant material at 65 °C until a constant mass was achieved. Leaf chlorophyll content was measured prior to harvest, representing the physiological maturity stage of the plants, using a portable chlorophyll meter (Konica Minolta, Tokyo, Japan).

### 2.8. Analysis of Micro and Macro Elements in Soil and Plants

Plant and soil samples were oven-dried in a forced-air drying system at 45–50 °C for 4–5 days to reach a constant weight. Total mineral element concentrations in plant tissues were determined following microwave-assisted acid digestion. Finely ground plant material was digested with concentrated nitric acid (HNO_3_) using a closed-vessel microwave digestion system. The resulting digests were analyzed for phosphorus (P), potassium (K), calcium (Ca), magnesium (Mg), iron (Fe), and zinc (Zn) via Inductively Coupled Plasma Optical Emission Spectrometry (ICP-OES, Thermo Fisher Scientific, Waltham, MA, USA) [34].

For soil samples, plant-available nutrient fractions were quantified using specific chemical extraction procedures prior to ICP-OES determination [35]. Available P was extracted using the sodium bicarbonate (Olsen) method. Exchangeable K, Ca, and Mg were extracted with 1 M ammonium acetate. Plant-available micronutrients, including Fe, Mn, Zn, and Cu, were extracted using the DTPA (diethylenetriaminepentaacetic acid) method. Total nitrogen (N) content in both plant and soil samples was determined via an automated nitrogen analyzer based on the Kjeldahl principle. Chloride (Cl^−^) concentrations were determined by titrimetric analysis.

### 2.9. Salt Tolerance Characterization

To confirm halotolerance, the isolates were tested for their ability to tolerate different salt concentrations on TSA plates containing 0%, 3%, 6%, 12%, 15% and 18% of NaCl and their growth was monitored for 10 days. Both strains exhibited sustained growth up to 18% of NaCl. These observations support their classification as halotolerant, although precise tolerance thresholds require further experimental validation [17].

### 2.10. Statistical Analysis

All experiments were performed using a completely randomized design. For both seed germination and greenhouse assays, each treatment included five independent plant units as biological replicates. The entire experiment was repeated three times under the same conditions to ensure reproducibility. Technical replicates were included where appropriate for analytical measurements. Prior to statistical analysis, data were tested for normality and homogeneity of variance. Differences among treatments were evaluated using one-way ANOVA, followed by Duncan’s multiple range test at a significance level of *p* < 0.05. Effect sizes (η^2^) were calculated to better quantify the magnitude of treatment effects, and 95% confidence intervals were estimated when relevant. Although measurements were not conducted under blinded conditions, all procedures followed standardized protocols to ensure consistency and minimize potential bias. In addition, Pearson correlation analysis was conducted to evaluate the relationships between soil physicochemical properties and plant growth parameters. Correlation coefficients (r) and corresponding *p*-values were calculated, and statistical significance was set at *p* < 0.05.

## 3. Results

### 3.1. Biochemical Characterization of Plant Growth-Promoting Traits

Biochemical characterization of plant growth-promoting (PGP) traits revealed that strain XE-15 exhibited superior performance compared with XR-18. Strain XR-18 showed positive reactions for indole-3-acetic acid (IAA) production and iron-chelating siderophore synthesis (Table 1). Although pellicle formation observed in NFb medium suggests a potential for diazotrophic activity, this observation alone is not sufficient to confirm biological nitrogen fixation. Therefore, additional confirmatory analyses, such as the acetylene reduction assay, are required to validate this trait, as previously recommended [36].

### 3.2. Phylogenetic Characterization and Taxonomic Placement

PCR amplification, nucleotide sequencing, and subsequent raw sequence assembly resulted in partial 16S rRNA gene sequences of 1532 bp for isolate XE-15 and 1067 bp for isolate XR-18. Comparative sequence analyses demonstrated that these sequences exhibited 100% nucleotide identity with complete query coverage to reference sequences of *Bacillus* and *Pseudomonas* species available in the GenBank database (Table 2).

To verify the validity of sequence concatenation for multilocus sequence analysis (MLSA), phylogenetic reconstructions were performed independently for each individual housekeeping gene. The resulting phylogenetic trees (Figures S1–S6) displayed largely congruent topologies, thereby supporting the assumption of concordant evolutionary histories among the selected loci. On this basis, concatenation of the housekeeping gene sequences was considered appropriate for MLSA-based phylogenetic inference.

For isolate XR-18, concatenation of four housekeeping markers (*rpoB*, *groEL*, *gyrA*, and *purH*) yielded a total sequence length of 2733 bp. These extended nucleotide datasets enabled more robust phylogenetic resolution and were deposited in the GenBank database under the accession numbers provided in (Table 2).

**Figure 1:**
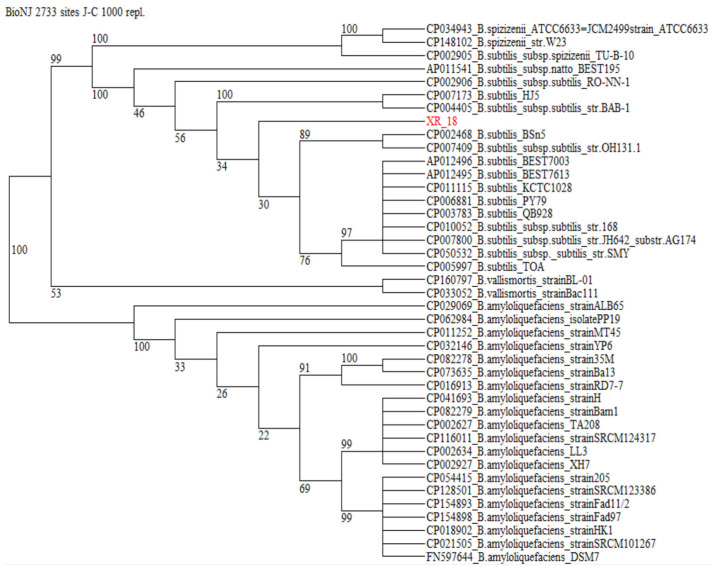
Phylogenetic relatedness of bacteria belonging to the Bacillus subtilis group (taxid: 653685) as reconstructed from concatenated *rpoB*, *groEL*, *gyrA*, and *purH* marker sequences. Terminal branches of the cladogram are labeled by GenBank accession number, genus, species and strain designations. The isolate under study is displayed in red. Numbers on internal branches indicate bootstrap support percentages. A concatenation of orthologous sequences from the related bacterium *B. subtilis* subsp. *spizizenii* served as an outgroup to root the tree.

**Figure 2:**
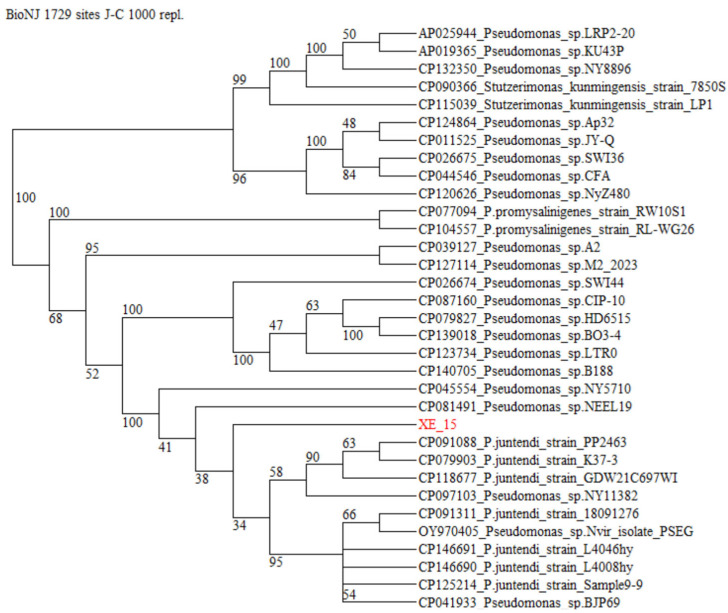
Phylogenetic relatedness of bacteria belonging to *Pseudomonas* (taxid: 286) as reconstructed from concatenated *rpoB* and *gyrB* marker sequences. Terminal branches of the cladogram are labeled by GenBank accession number, genus, species and strain designations. The isolate under study is displayed in red. Numbers on internal branches indicate bootstrap support percentages. A concatenation of orthologous sequences from the related bacterium *Stutzerimonas kunmingensis* served as an outgroup to root the tree.

The reference dataset retrieved for phylogenetic reconstruction comprised 40 annotated *Bacillus* strains and 32 *Pseudomonas* strains. In the NJ phylogeny generated for the Bacillus dataset from a concatenation of the four MLSA marker sequences (Figure 1), the clades representing the species *Bacillus subtilis* appeared well separated from *B. amyloliquefaciens* and *B. vallismortis*, receiving 100% bootstrap support. The isolate XR-18 was firmly placed within this *B. subtilis* clade, even if bootstrap support for the internal sub-clade of *B. subtilis* subspecies and the concomitant delineation of the *B. subtilis* subsp. subtilis appeared low. In the NJ phylogeny of *Pseudomonas* dataset from a concatenation of *rpoB* and *gyrB* sequences (Figure 2), the species *P. juntendi* and *P. promysalinigenes* appeared well separated, receiving not less than 99% bootstrap support. XE-15 was placed within the clade containing both unspecified species within the *Pseudomonas* genus (*Pseudomonas* sp.) and *P. juntendi* supported with 100% bootstrap support. However, XE-15 was located to an internal sub-clade of the *P. juntendi* clade, even if bootstrap support for this clade and the concomitant delineation of the species *Pseudomonas* sp. appeared low at the double-marker level.

ML phylogenetic reconstructions were also conducted as a complementary approach. The resulting ML trees (Figures S7 and S8) revealed comparable topologies to the BioNJ trees, lending further support to the phylogenetic placement of isolates XE-15 and XR-18.

Given the low bootstrap support for internal sub-clades, a definitive species-level assignment was deferred in favor of a genus-level classification (*Pseudomonas* sp. and *Bacillus* sp.) to maintain taxonomic rigor.

### 3.3. In Vitro Seed Germination Assays

**Figure 3:**
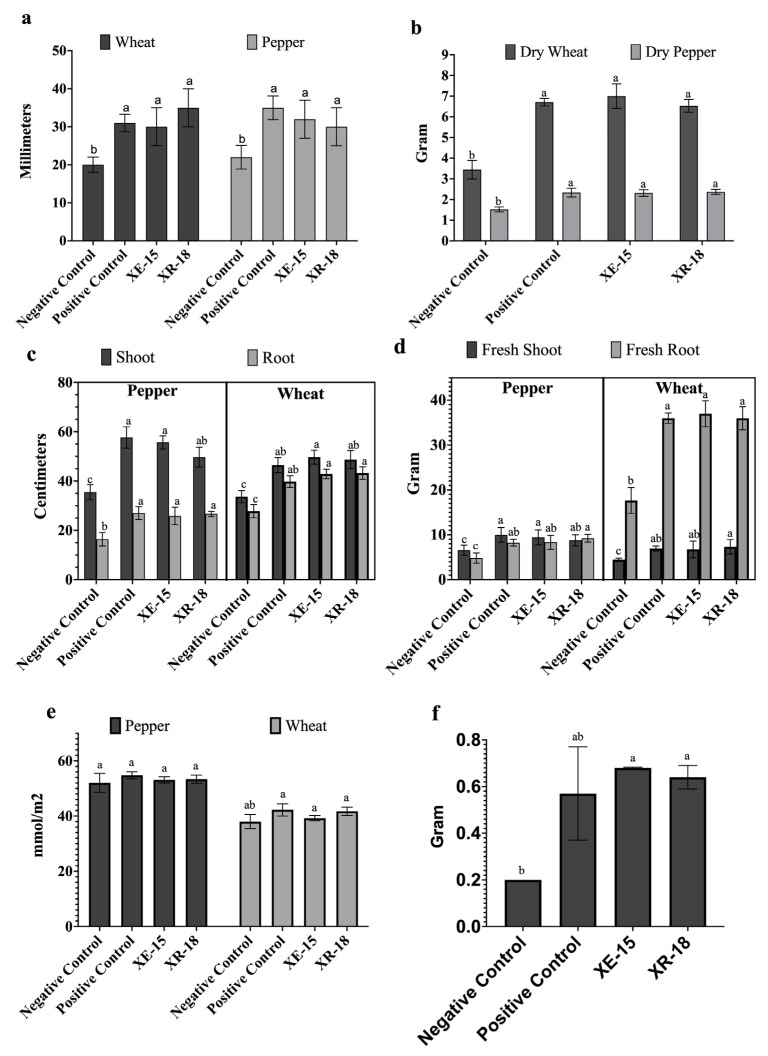
Effects of bacterial treatments on (**a**) seedling length (germination stage), (**b**) dry weights of seedlings, (**c**) shoot and root lengths, (**d**) fresh shoot and root weights, (**e**) chlorophyll content, and (**f**) dry fruit weights of pepper plants. All parameters in panels (**a**–**e**) were evaluated for both wheat and pepper, while panel (**f**) represents pepper only. Bars with same color and different letters differ significantly according to Duncan’s multiple range test (*p* < 0.05).

The 51 bacterial isolates initially obtained were subjected to a multi-stage screening process to identify the most effective candidates. In the primary phase, isolates were prioritized based on their multi-functional biochemical PGP profiles; those displaying inconsistent or singular growth-promoting traits were excluded from further study. Following this qualitative assessment, the most promising candidates were evaluated in seed germination assays to quantify their direct impact on early plant development. Among the initial pool, strains XE-15 and XR-18 were ultimately selected as they significantly enhanced both wheat and pepper seedling growth in vitro compared to the negative control. Specifically, while XE-15 was more effective in enhancing pepper seedling growth, XR-18 demonstrated superior performance in promoting wheat seedling length (Figure 3a).

### 3.4. Enhancement of Plant Growth and Productivity in Sandy Soil

The synthetic soil used for in vivo assays was previously characterized with various physicochemical analysis. According to physicochemical analyses, the soil is considered as sandy, with a bulk density of 1.37 g cm^−3^ typically for loamy sand or other sandy soils, suggesting a moderately compacted soil. Macro- and microelement content shows low nutrient availability, which is supported by low EC and C/N ratio. The initial culturable bacterial population of the sandy soil averaged 1.1 × 10^5^ CFU g^−1^. After the three inoculation events, total counts stabilized between 6 × 10^6^ and 1 × 107 CFU g^−1^, indicating a 1–1.5-log increase and suggesting successful establishment of the applied strains.

**Figure 4:**
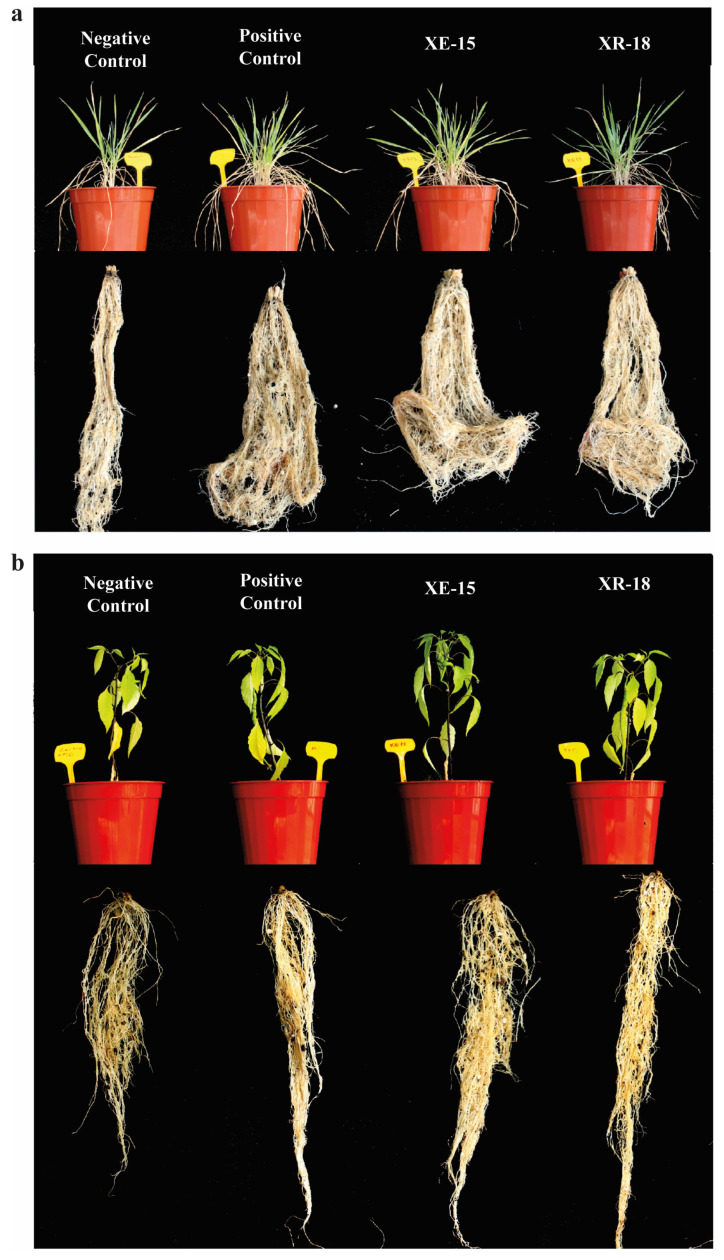
Photographs of (**a**) wheat and (**b**) pepper plants showing both potted growth and washed roots after harvest. These represent typical samples from the experimental groups.

The effect of sandy soil inoculation with rhizobacterial strains on wheat and pepper plant growth was assessed through different parameters after 120 days of culture in pots, under greenhouse conditions. According to Duncan’s homogenous groups in Figure 3, t inoculation with the strains resulted in a measurable increase in the length and fresh weight of wheat shoots and roots compared to the negative control. They exhibited a comparable growth-promoting effect to the positive control treated with a commercial solution of *B. megaterium* M3 (Figure 4) Statistical analysis of plant’s length and fresh weight showed significant effect of soil inoculation with bacterial strains in improving pepper plant growth (Figure 3). A significant enhancement of plant growth was observed in plants treated with bacterial strains as dry mass means of wheat and pepper plants were almost double the negative control. Fruit production was significantly higher than the negative control and statistically equivalent to the positive control in pepper plants treated with XE-15 (0.68 g/fruit) and XR-18 (0.64 g/fruit) strains than in positive (0.57 g/fruit) and negative (0.2 g/fruit) control trials.

### 3.5. Macro- and Microelement Accumulation in Plants

Macro- and micronutrient concentrations in the leaves and roots of wheat and pepper plants are summarized in Table 3 and Table 4. As shown in Table 3, bacterial inoculation with strains XE-15 and XR-18 resulted in a marked enhancement of nutrient accumulation in wheat compared with the control treatments, particularly with respect to nitrogen (N), phosphorus (P), magnesium (Mg), and zinc (Zn). Among the tested strains, XR-18 induced the greatest increases in iron (Fe; 198.65 mg kg^−1^) and phosphorus (P; 7.98 mg kg^−1^). In contrast, plants inoculated with strain XE-15 exhibited the highest concentrations of nitrogen (1.08%), magnesium (4.11 mg kg^−1^), zinc (102.3 mg kg^−1^), and calcium (Ca; 36.56 mg kg^−1^).

Both positive and negative control treatments generally resulted in lower concentrations of several key elements, including phosphorus, iron, and zinc. Notably, the Bacillus megaterium M3 strain used as the positive control frequently exhibited comparatively lower nutrient accumulation in wheat tissues relative to the XE-15 and XR-18 treatments, suggesting a strong plant growth-promoting effect of the latter strains under the experimental conditions.

According to (Table 4), XE-15 was likely to be the most effective bacterial treatment for enhancing several macro- and micro-nutrient levels in leaves and roots of pepper, especially for phosphorus (19.90 mg kg^−1^), potassium (142.67 mg kg^−1^), and zinc (317.3 mg kg^−1^), while treatment XR-18 showed benefits in specific elements such as calcium (29.76 mg kg^−1^) and chlorine (19.44 mg kg^−1^). Interestingly, negative control (NC) showed the highest levels of iron (150.18 mg kg^−1^) and calcium (35.58 mg kg^−1^). Positive control (PC) generated intermediate results, with moderate increases in phosphorus (16.01 mg kg^−1^), zinc (216.4 mg kg^−1^), and sodium (4.77 mg kg^−1^).

### 3.6. Impact on Soil Quality and Physicochemical Properties

The effects of the different treatments on soil physicochemical properties, relative to the initial soil conditions prior to cultivation, are presented in (Table 5). Overall, bacterial inoculation improved soil properties relative to both the baseline soil (before plantation) and the uninoculated negative control (NC), with strain XR-18 showing the strongest positive effects. Soil pH values indicated slightly alkaline conditions in the NC treatment (pH 7.61). In contrast, soils subjected to bacterial inoculation exhibited a tendency toward mild acidification, with the lowest pH recorded in the positive control treatment (pH 6.68). In addition, bacterial treatments were associated with a reduction in electrical conductivity (EC) values, suggesting a decrease in overall soil salinity. This reduction in EC, together with the slight acidification observed in inoculated soils, is likely to enhance the bioavailability of micronutrients such as iron (Fe) and zinc (Zn).

Analysis of physical parameters revealed that all bacterial treatments (PC, XE-15, and XR-18) resulted in a statistically significant increase in water-holding capacity (WHC) compared with the negative control (NC) (*p* < 0.05). Specifically, WHC values were 8.47 ± 0.31% in the NC treatment, whereas values increased to 10.33 ± 0.10%, 9.80 ± 0.14%, and 10.00 ± 0.15% in the PC, XE-15, and XR-18 treatments, respectively. These increases in WHC were observed despite only marginal numerical reductions in soil bulk density across the treatments.

From a chemical perspective, bacterial inoculation significantly enhanced the availability of several essential soil nutrients relative to the negative control. Notably, zinc (Zn) availability was significantly increased (*p* < 0.05) in soils treated with XE-15 (6.48 ± 0.97 mg kg^−1^) and XR-18 (9.37 ± 1.21 mg kg^−1^). In addition, iron (Fe) availability was significantly elevated in the PC and XR-18 treatments compared with the NC, indicating a positive effect of bacterial inoculation on micronutrient mobilization in the soil XR-18 treatments.

### 3.7. Correlation Between Soil Properties and Plant Performance

Pearson correlation analysis revealed strong positive associations between soil micronutrient availability and plant nutrient uptake. Specifically, available Fe and Zn levels in the soil were positively correlated with their respective concentrations in plant tissues, with r values ranging from 0.62 to 0.81 (*p* < 0.05). Furthermore, soil water-holding capacity exhibited a significant positive correlation with total plant biomass (r ≈ 0.70), suggesting that the improvement in soil physical structure directly contributed to enhanced crop productivity in the nutrient-deficient sandy substrate.

## 4. Discussion

### 4.1. Global Soil Degradation and the Strategic Role of PGPR

Globally, an estimated 20–30 billion metric tons of soil are lost annually due to erosion and degradation processes [37]. This trend is expected to intensify as climate change interacts with socio-economic pressures, particularly in arid and semi-arid regions. Soil degradation therefore constitutes a growing threat to food security, ecosystem biodiversity, water availability, and the long-term economic stability of nations. In this context, the implementation of sustainable soil management practices within conservation strategies, together with addressing the underlying drivers of soil degradation, is essential to safeguard soil resources for future generations.

Within its agro-environmental framework, the European Union identifies soil fertility as a cornerstone of soil health. The rehabilitation of marginal or abandoned lands not only mitigates erosion and compaction but also contributes to the restoration of soil fertility. In line with this perspective, the EU Soil Observatory recognizes soil microbiome diversity as a strategic priority under the European Green Deal [38]. In this regard, halotolerant plant growth-promoting rhizobacteria (PGPR) have attracted increasing attention due to their ability to function under conditions of salinity, alkalinity, and nutrient limitation, while simultaneously enhancing nutrient availability, soil structure, plant growth, salinity tolerance, and biocontrol capacity [39]. Leveraging these attributes, the present study evaluated two newly isolated halotolerant bacterial strains, XE-15 and XR-18, for their capacity to support wheat (*Triticum aestivum* L.) and pepper (*Capsicum annuum* L.) growth in unfertilized, nutrient-poor sandy soil, an edaphic environment inherently characterized by low nutrient availability and limited water-holding capacity (WHC) [40]. Inoculation with these strains resulted in marked improvements in nutrient availability and overall soil quality.

### 4.2. Multifunctional Growth-Promoting Traits and Taxonomic Resolution

Among the 51 bacterial isolates initially screened, XE-15 and XR-18 demonstrated the most pronounced enhancement of seedling growth under in vitro conditions. Notably, these strains appeared to operate through distinct growth-promoting mechanisms and exhibited differential efficacy depending on the plant species. Strain XE-15 displayed a broader spectrum of PGPR traits, including nitrogen fixation, phosphate and potassium solubilization, indole-3-acetic acid (IAA) production, and siderophore synthesis, suggesting a multifunctional growth-promotion strategy. In contrast, XR-18, while lacking several nutrient-mobilizing traits, exhibited pronounced IAA and siderophore production, which may sufficiently explain its growth-promoting effects on wheat through hormonal regulation and enhanced iron acquisition. Although XR-18 did not demonstrate phosphate solubilization under in vitro conditions, its capacity to enhance phosphorus uptake in wheat indicates the involvement of indirect mechanisms. Specifically, 1-aminocyclopropane-1-carboxylate (ACC) deaminase activity and IAA production likely contributed to modifications in root architecture, thereby increasing root surface area and nutrient uptake efficiency, while protease activity may have facilitated the mineralization of organic phosphorus pools in the soil. Siderophore-producing bacteria are widely recognized as an important class of PGPR due to their dual role in disease suppression and plant growth enhancement [41]. In particular, *Pseudomonas* species are well-known for their capacity to suppress soil-borne pathogens and to modulate plant immune responses through complex interactions with root exudates [42,43].

Initial taxonomic identification based on partial 16S rRNA gene sequences placed isolates XE-15 and XR-18 within the genera *Pseudomonas* and *Bacillus*, respectively; however, species-level resolution could not be confidently achieved through 16S rRNA alone. Multilocus sequence analysis (MLSA) using conserved housekeeping genes (e.g., *rpoB*, *gyrB*, *groEL*, *gyrA*, *purH*) is known to provide higher phylogenetic resolution and more robust evolutionary inference [29,30,31]. Although concatenated phylogenetic analyses grouped XE-15 and XR-18 with *P. juntendi* and *B. subtilis*, respectively, limited internal sub-clade support suggests that further taxonomic characterization is warranted.

While MLSA using conserved housekeeping genes provides significantly higher resolution than 16S rRNA analysis alone, the observed low bootstrap support for specific internal sub-clades indicates that sequence-based markers may still be insufficient for definitive species-level delimitation within these complex genera. To maintain taxonomic rigor and avoid premature classification, the isolates XE-15 and XR-18 are strictly referred to as *Pseudomonas* sp. and *Bacillus* sp. throughout this study. We acknowledge that achieving a formal species assignment will require the application of genome-scale metrics, such as Average Nucleotide Identity (ANI) and digital DNA–DNA hybridization (dDDH), which remain a priority for our future investigations.

### 4.3. Plant-Strain Specificity and Comparative Efficacy in Greenhouse Assays

The in vitro observations were consistent with the improved growth performance of wheat and pepper plants under greenhouse conditions. Both XE-15 and XR-18 significantly enhanced plant growth parameters relative to the negative control (NC), although a degree of plant-strain specificity was evident. Strain XR-18 was particularly effective in promoting wheat growth, especially with respect to biomass accumulation, root development, and chlorophyll content, whereas XE-15 exerted a stronger effect on pepper growth, notably in shoot and root elongation and biomass production. Such host-dependent responses are well documented, as PGPR efficacy is often influenced by plant genotype [44]. Host specificity reflects the preferential interaction between microbial species and particular plant genotypes, leading to variable outcomes in growth promotion and disease suppression [45]. In both wheat and pepper experiments, XE-15 and XR-18 generally performed comparably *B. megaterium* M3 [32], particularly in terms of root growth, shoot length, and fresh biomass. The observed performance of the newly isolated strains suggests that they may be more efficient under nutrient-deficient sandy soil conditions. This advantage is likely linked to their halotolerant origin, which enables successful establishment and functionality under the mild salinity indicated by elevated sodium and chloride levels in the soil. Previous ecological studies have similarly demonstrated that PGPR originating from stress-prone environments are often more effective in enhancing plant tolerance to specific abiotic stresses [46,47,48].

### 4.4. Nutrient Acquisition and Biofortification in Nutrient-Poor Soil

The synthetic soil used in the pot experiments was classified as sandy but exhibited moderate compaction, with a bulk density of 1.37 g cm^−3^. It was characterized by low concentrations of essential macro- and micronutrients, including nitrogen (0.30%), iron (4.13–5.00 mg kg^−1^), and phosphorus (17.62 mg kg^−1^). For comparison, winter wheat typically requires approximately 200 kg N ha^−1^, 24 kg P ha^−1^, and 210 kg K ha^−1^ to achieve a target grain yield [49]. Among solanaceous crops, pepper exhibits particularly high demands for potassium (40%) and nitrogen (31%) relative to total nutrient uptake [50].

Under these challenging edaphic conditions, both XE-15 and XR-18 significantly enhanced nutrient uptake and increased plant dry mass. However, XE-15 was slightly more effective in improving pepper yield, as evidenced by significantly higher zinc (Zn) concentrations and numerically increased phosphorus (P) and potassium (K) levels in pepper tissues. Conversely, XR-18 was more effective in enhancing iron (Fe) and phosphorus uptake in wheat. The capacity of XR-18 to enhance phosphorus accumulation, despite its negative in vitro solubilization result, suggests an indirect growth-promotion strategy rather than direct mineral dissolution. This effect may be attributed to the strain’s ability to produce IAA, which is associated with root elongation and an increase in the overall surface area available for nutrient absorption. Such morphological shifts are clearly visible in the harvested root samples (Figure 4), supporting the hypothesis that enhanced nutrient uptake is a function of modulated root–soil contact. Additionally, the detected protease activity in XR-18 (Table 1) may facilitate the mineralization of soil organic matter, potentially releasing associated organic phosphorus pools into plant-available forms. Previous studies have documented that inoculation with plant growth-promoting bacteria enhances nutrient availability and uptake in cereals, leading to increases in dry biomass and nutrient content compared with uninoculated controls [51,52,53]. In wheat, bacterial inoculants have been shown to elevate N, P, and K levels in both soil and plant tissues [52]. Similarly, zinc-solubilizing bacteria have been reported recently to improve Zn biofortification and plant biomass [51]. In particular, the role of PGPB in facilitating micronutrient uptake, especially Zn, by modifying root architecture and nutrient mobilization has been emphasized in the recent literature [53,54].

### 4.5. Impact on Soil Physical Properties and Future Ecological Perspectives

In addition to plant responses, bacterial inoculation improved soil quality by reducing bulk density, increasing water-holding capacity, and enhancing the availability of key nutrients. These effects are likely linked to the production of extracellular polymeric substances (EPS) by *Bacillus* and *Pseudomonas* species, which contribute to the formation of stable soil aggregates and the alleviation of soil compaction [55]. Furthermore, the slight acidification observed in bacterially treated soils likely facilitated altered nutrient dynamics and bioavailability. Overall, the newly isolated halotolerant strains XE-15 and XR-18 show strong potential for supporting wheat and pepper cultivation in nutrient-poor sandy soil by enhancing nutrient availability and soil quality while reducing dependence on chemical fertilizers. Nevertheless, it is important to acknowledge that the greenhouse experiments were conducted using a simplified artificial soil substrate. Although this approach allowed controlled assessment, it does not fully capture the complexity of natural soils, which harbor diverse microbial communities that may interact with or compete against introduced strains [56]. For instance, the impact of PGPR inoculation in wheat strongly depends on interactions between introduced strains and the native soil microbial community [57]. Consequently, the observed plant growth-promoting effects should be considered preliminary, and further validation under field conditions using microbiologically complex soils is required to confirm the efficacy and ecological adaptability of XE-15 and XR-18.

The use of a sterile, simplified substrate represents a major limitation, as it excludes native microbial communities and associated ecological interactions. Therefore, the observed colonization success does not necessarily reflect competitive persistence or functional dominance under field conditions. Moreover, while these results are promising, certain functional indicators of soil quality, such as soil enzyme activities (e.g., urease, phosphatase) and soil microbial biomass, were not quantified in the current study. The absence of these measurements represents a limitation in fully characterizing the biological drivers of soil health improvement. Future investigations utilizing metagenomic sequencing and enzymatic profiling will be essential to validate the in situ expression of PGP traits and to confirm the functional efficacy of XE-15 and XR-18 within microbiologically complex natural soils.

While the PGPR strains evaluated herein were isolated from hypersaline Saharan environments, this study primarily investigated their performance within a non-saline, nutrient-deficient sandy matrix. This experimental framework was designed to elucidate the capacity of halotolerant isolates to confer cross-stress protection, particularly under conditions of limited nutrient and water availability. It is, however, pertinent to acknowledge that the functional efficacy of these strains in situ may be modulated by complex interactions with indigenous microbiota and fluctuating salinity gradients. Consequently, future research encompassing multi-environmental field trials spanning both saline and non-saline conditions will be indispensable to fully ascertain the ecological versatility and adaptive breadth of XE-15 and XR-18.

## 5. Conclusions

The present study provides initial evidence that the halotolerant plant growth-promoting rhizobacterial strains XE-15 and XR-18 can enhance plant growth and improve key soil physicochemical parameters in a nutrient-deficient artificial substrate. These findings support their potential application as biofertilizers in low-fertility soils. However, future investigations should incorporate experiments conducted in natural soils to assess their performance within complex native microbial communities and to validate their efficacy under realistic field conditions. Although preliminary phylogenetic analyses tentatively assigned XE-15 and XR-18 to the genera *Pseudomonas* and *Bacillus*, respectively, additional taxonomic characterization is required to achieve definitive species-level identification. Both strains exhibited distinct and complementary effects on wheat and pepper growth, underscoring their promise as sustainable bioinoculants. Future research should prioritize (i) whole-genome sequencing to confirm taxonomic identity and explore functional gene repertoires, (ii) mechanistic validation of plant–microbe interactions through root phenotyping and soil enzymatic profiling, and (iii) multi-location field trials across different soil types and climatic conditions. Additionally, co-inoculation strategies combining XE-15 (nutrient mobilization) and XR-18 (hormonal modulation and stress tolerance) represent a promising avenue for optimizing biofertilizer formulations.

## Figures and Tables

**Table 1:** Biochemical characterization of plant growth-promoting traits for isolates XE-15 and XR-18.

Strains Codes	M3	XE-15	XR-18
Species	*B. megaterium*	*Pseudomonas* sp.	*Bacillus* sp.
N-fixation activity	−	+	−
Phosphate solubilization	+	+	−
Potassium Solubilization	+	+	−
Zinc solubilization	−	+	−
Iron siderophore production	−	+	+
HCN production	−	−	−
IAA production	+	+	+
ACC deaminase	−	−	+
Amylase	−	−	+
Protease	+	−	+
Lipase	−	−	−
Cellulase	−	−	−

N.B. (+) to designate the presence of the activity/production; (−) to designate the absence of the activity/production.

**Table 2:** Molecular identification and GenBank accession numbers for isolates XE-15 and XR-18.

Isolate	Housekeeping Gene	Sequence Length	Accession Number	16S rRNA 100% of Similarity and Coverage
XE-15	*16S*	1532 bp	PV400807	*P. juntendi* *Pseudomonas* sp. *P. promysalinigenes*
	*rpoB*	1047 bp	PX933507	
	*gyrB*	888 bp	PX933508	
XR-18	*16S*	1067 bp	PV400808	*B. amyloliquefaciens* *B. vallismortis* *B. subtilis* subsp. *subtilis* *B. velezensis*
	*rpoB*	813 bp	PX933303	
	*groEL*	624 bp	PX933504	
	*gyrA*	669 bp	PX933505	
	*purH*	627 bp	PX933506	

**Table 3:** Macro- and microelement concentrations in wheat leaves and roots. Values are means ± SD. Different letters within a column indicate significant differences according to Duncan’s multiple range test (*p* < 0.05).

(mg kg^−1^)	NC	PC	XE-15	XR-18
N (%)	0.415 ± 0.050 a	0.84 ± 0.07 b	1.08 ± 0.088 c	0.86 ± 0.054 b
P (mg kg^−1^)	6.27 ± 1.04 b	2.92 ± 0.96 a	6.87 ± 0.85 b	7.98 ± 1.32 b
K (mg kg^−1^)	37.84 ± 6.32 a	41.78 ± 9.37 a	40.87 ± 7.33 a	39.19 ± 11.04 a
Fe (mg kg^−1^)	80.17 ± 18.52 b	37.73 ± 7.73 a	110.48 ± 26.33 b	198.65 ± 47.64 c
Mg (mg kg^−1^)	2.05 ± 1.02 a	2.56 ± 0.56 a	4.11 ± 0.78 a	3.17 ± 0.88 a
Zn (mg kg^−1^)	74.80 ± 5.88 b	51.50 ± 9.38 a	102.3 ± 13.63 c	89.86 ± 5.31 c
Ca (mg kg^−1^)	15.99 ± 2.19 a	10.94 ± 3.89 a	36.56 ± 6.12 c	22.66 ± 4.23 b
Na (mg kg^−1^)	4.76 ± 0.98 a	5.45 ± 1.10 a	4.55 ± 1.30 a	5.33 ± 0.91 a
Cl (mg kg^−1^)	27.89 ± 4.12 a	21.75 ± 3.22 a	23.56 ± 3.33 a	22.35 ± 4.02 a

**Table 4:** Effects of different bacterial treatments on macro- and microelement composition of leaves and roots of pepper (mg kg^−1^). Values are means ± SD (*n* = 5). Different letters within a column indicate significant differences according to one-way ANOVA followed by Duncan’s multiple range test (*p* < 0.05).

(mg kg^−1^)	NC	PC	XE-15	XR-18
N %	1.25 ± 0.04 b	1.50 ± 0.03 c	1.02 ± 0.055 a	1.28 ± 0.051 b
P (mg kg^−1^)	10.08 ± 3.63 a	16.01 ± 3.73 a	19.90 ± 4.74 a	14.17 ± 3.77 a
K (mg kg^−1^)	115.44 ± 22.25 a	71.60 ± 25.31 a	142.67 ± 17.81 a	82.79 ± 23.59 a
Fe (mg kg^−1^)	150.18 ± 36.04 a	93.90 ± 21.67 a	125.84 ± 29.83 a	90.07 ± 21.43 a
Mg (mg kg^−1^)	8.74 ± 1.11 a	8.57 ± 1.83 a	9.57 ± 1.89 a	6.70 ± 1.10 a
Zn (mg kg^−1^)	207.93 ± 18.94 a	216.40 ± 18.95 a	317.30 ± 10.61 b	275.90 ± 18.89 a
Ca (mg kg^−1^)	35.58 ± 11.16 a	30.58 ± 11.83 a	25.61 ± 9.13 a	29.76 ± 10.61 a
Na (mg kg^−1^)	5.22 ± 0.84 a	4.77 ± 0.41 a	5.51 ± 0.31 a	3.38 ± 0.49 a
Cl (mg kg^−1^)	18.58 ± 3.80 a	20.87 ± 5.31 a	15.70 ± 3.50 a	19.44 ± 3.03 a

**Table 5:** Physico-chemical compositions of soil before and after wheat culture under different treatments. Values are means ± SD (*n* = 5). Different letters within a column indicate significant differences according to one-way ANOVA followed by Duncan’s multiple range test (*p* < 0.05).

	Initial	NC	PC	XE-15	XR-18
Bulk density (g cm^−3^)	1.37 ± 0.039 a	1.36 ± 0.022 a	1.32 ± 0.020 a	1.35 ± 0.020 a	1.32 ± 0.020 a
pH	7.17 ± 0.072 ab	7.61 ± 0.086 a	6.68 ± 0.067 c	6.97 ± 0.071 ab	6.72 ± 0.067 c
EC (µS cm^−1^)	274.30 ± 22.43 d	174.30 ± 12.40 c	127.80 ± 11.73 b	72.50 ± 10.01 a	156.60 ± 15.51 c
WHC * (%)	7.43 ± 1.15 a	8.47 ± 0.31 a	10.33 ± 0.10 c	9.80 ± 0.14 b	10.00 ± 0.15 b
Fe (mg kg^−1^)	4.13 ± 0.90 a	50.00 ± 10.10 b	432.80 ± 82.50 d	204.00 ± 49.80 c	370.00 ± 73.20 d
Mg (mg kg^−1^)	349.00 ± 23.92 a	549.00 ± 45.92 b	536.00 ± 41.88 b	455.00 ± 39.40 b	480.00 ± 49.40 b
Zn (mg kg^−1^)	3.52 ± 0.63 a	4.42 ± 0.67 a	4.57 ± 0.61 a	6.48 ± 0.97 b	9.37 ± 1.21 c
Ca (mg kg^−1^)	5.03 ± 0.55 a	4.45 ± 0.40 a	4.79 ± 0.41 a	4.84 ± 0.81 a	4.49 ± 0.39 a
Na (mg kg^−1^)	234.44 ± 31.17 a	204.10 ± 31.62 a	188.50 ± 22.28 a	190.30 ± 38.55 a	262.50 ± 39.38 a
Cl (mg kg^−1^)	216.12 ± 42.20 a	285.90 ± 58.10 a	348.55 ± 61.70 a	215.00 ± 42.00 a	529.00 ± 115.80 b
K (mg kg^−1^)	135.32 ± 11.80 a	152.10 ± 12.10 a	135.10 ± 13.80 a	126.30 ± 10.10 a	136.00 ± 10.80 a
P (mg kg^−1^)	17.60 ± 2.31 b	16.20 ± 1.94 b	15.90 ± 1.51 b	11.30 ± 1.96 a	16.40 ± 1.97 b
Total N (%)	0.19 ± 0.05 a	0.30 ± 0.04 a	0.21 ± 0.02 a	0.61 ± 0.03 c	0.49 ± 0.04 b

(*) Water holding capacity.

## Data Availability

The original contributions presented in this study are included in the article/Supplementary Material. Further inquiries can be directed to the corresponding author. The 16S rRNA and MLSA sequences have been de-posited in the GenBank database (accession numbers will be provided upon publication).

## Supplementary Materials

The following supporting information can be downloaded at: https://www.mdpi.com/article/10.3390/microorganisms14051120/s1. Figure S1: Individual phylogenetic tree for the *gyrB* gene of *Pseudomonas* sp. XE-15. Figure S2: Individual phylogenetic tree for the *rpoB* gene of *Pseudomonas* sp. XE-15. Figure S3: Individual phylogenetic tree for the *groEL* gene of *Bacillus* sp. XR-18. Figure S4: Individual phylogenetic tree for the *gyrA* gene of *Bacillus* sp. XR-18. Figure S5: Individual phylogenetic tree for the *purH* gene of *Bacillus* sp. XR-18. Figure S6: Individual phylogenetic tree for the *rpoB* gene of *Bacillus* sp. XR-18. Figure S7: Maximum likelihood (ML) phylogenetic reconstruction for isolate *Pseudomonas* sp. XE-15. Figure S8: Maximum likelihood (ML) phylogenetic reconstruction for isolate *Bacillus* sp. XR-18.
