# Supplementary figures and images for: Halotolerant Rhizobacteria from Phragmites Communis: A Controlled Proof-of-Concept for Crop Improvement in Degraded Sandy Soils
Source: Microorganisms. 2026 May 14;14(5):1120. doi: 10.3390/microorganisms14051120 (PMC13209630; doi:10.3390/microorganisms14051120)

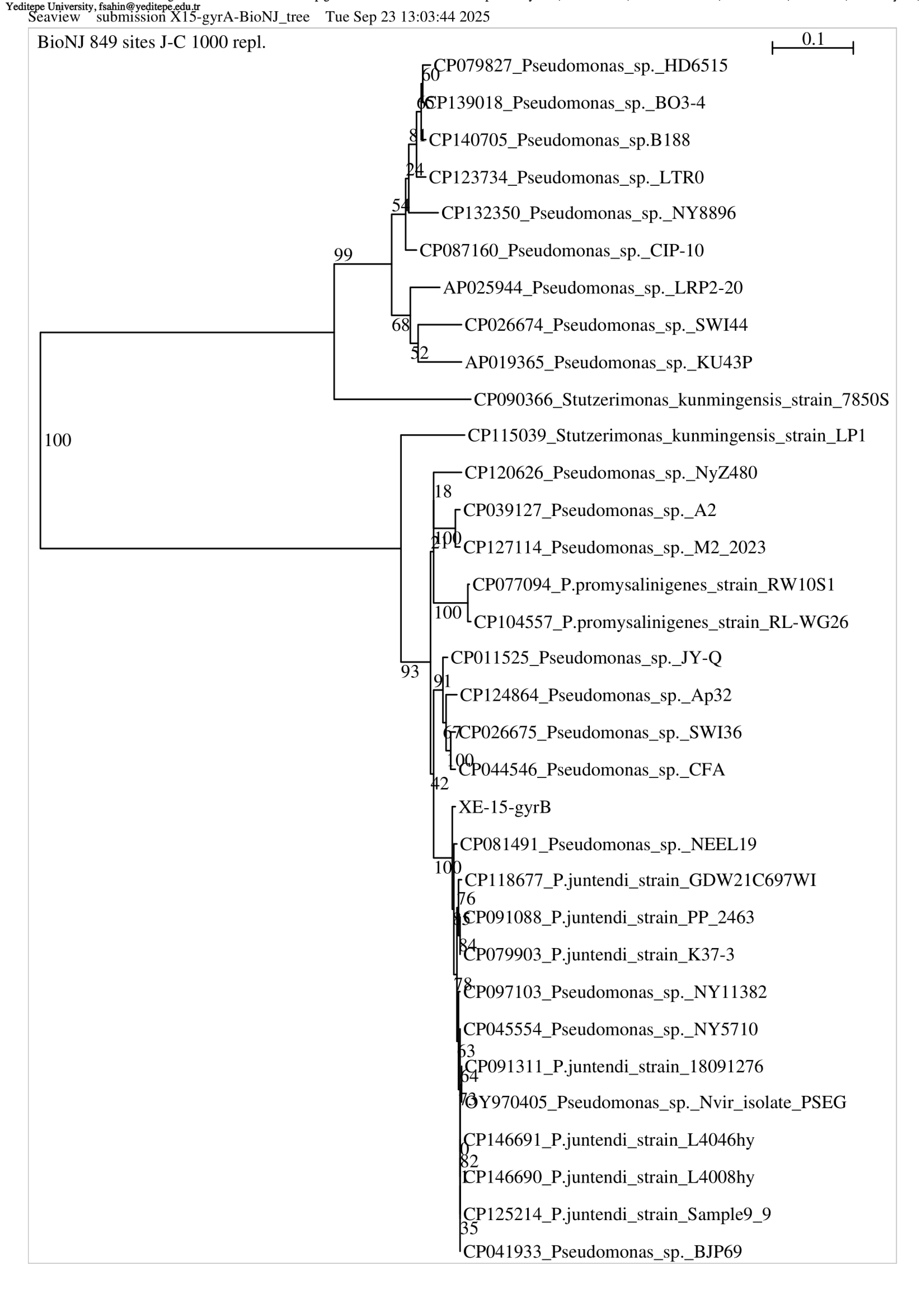

Supplement: Supplementary file 1 [file microorganisms-14-01120-s001.zip › Figure S1.tif]

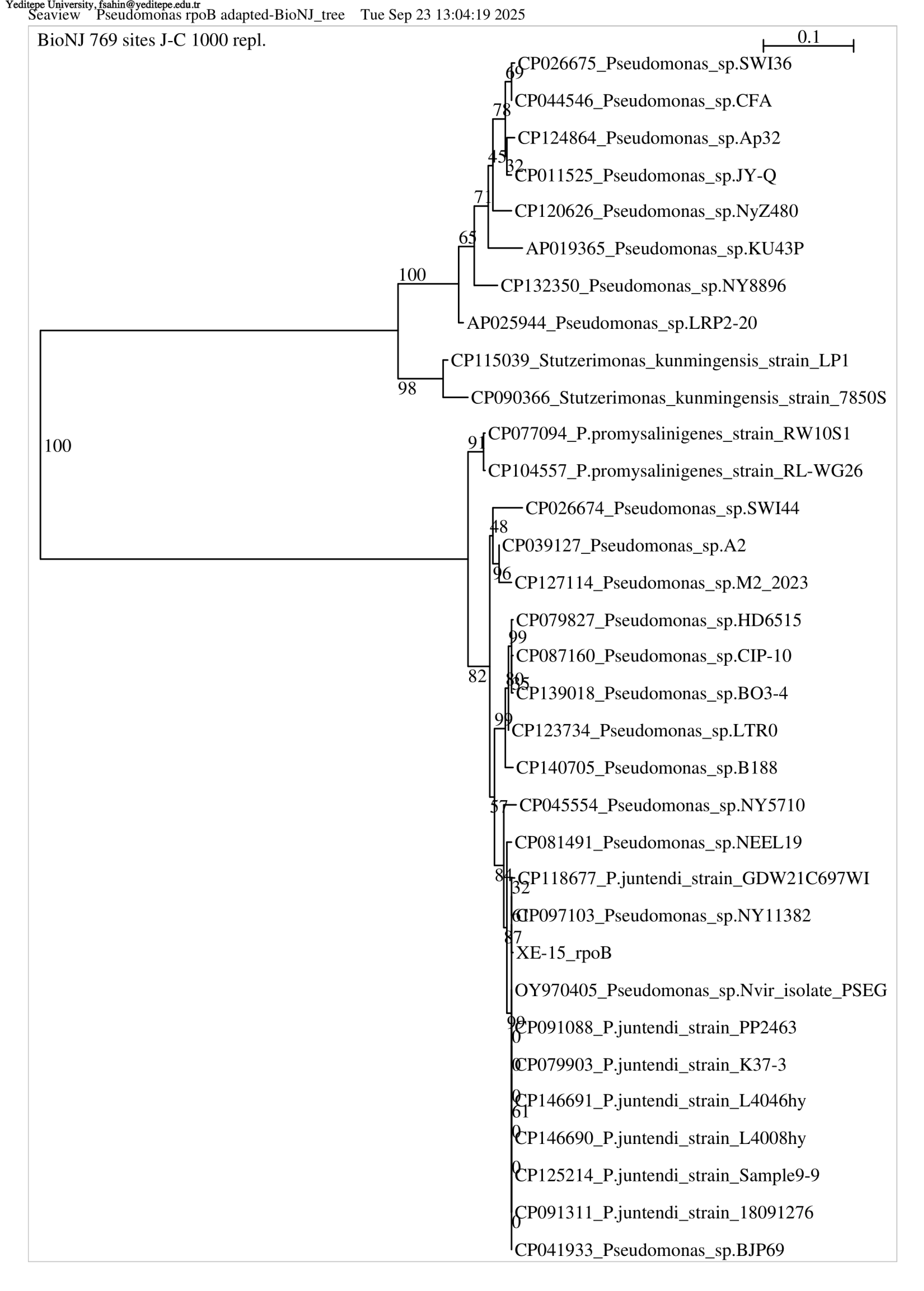

Supplement: Supplementary file 1 [file microorganisms-14-01120-s001.zip › Figure S2.tif]

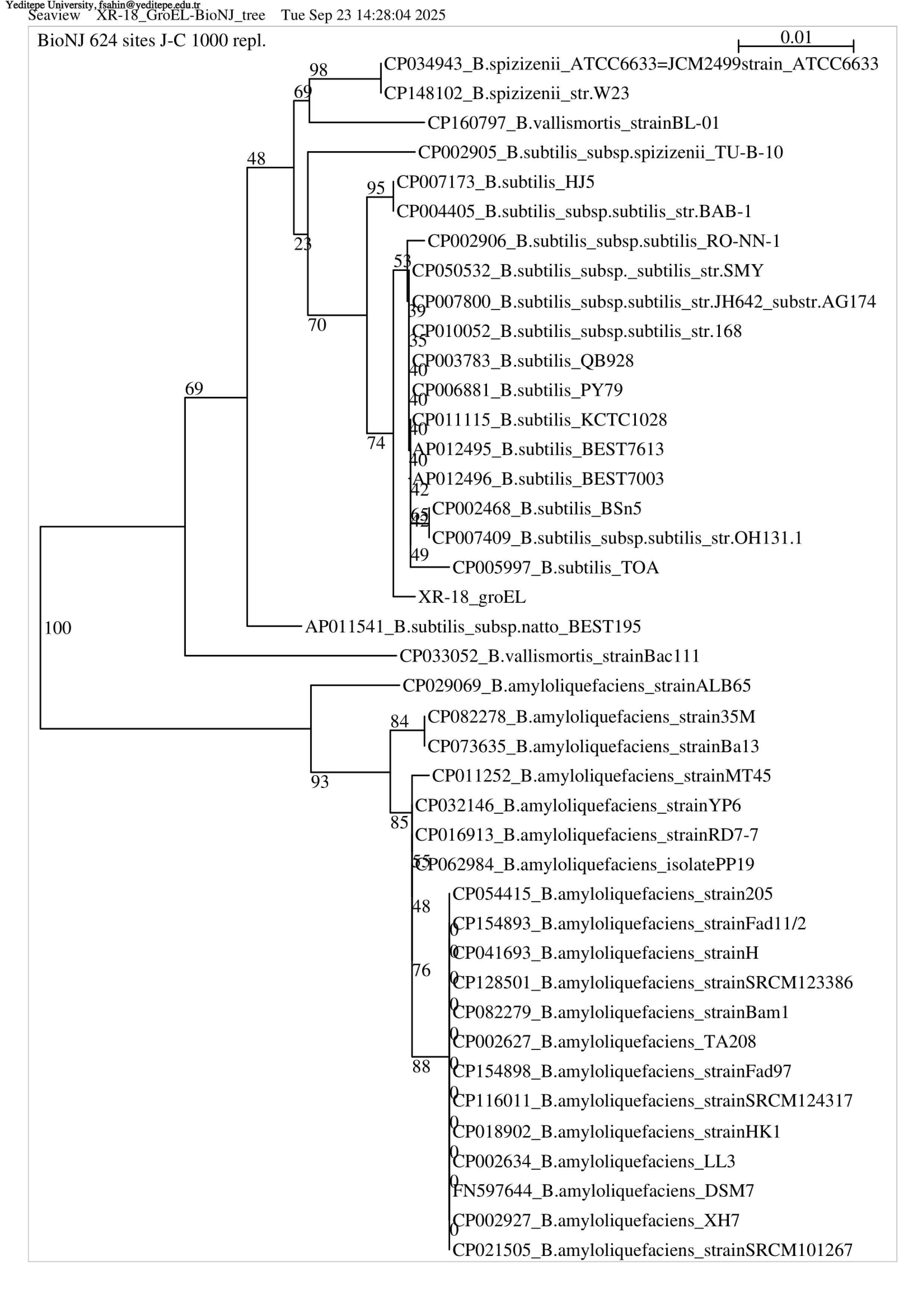

Supplement: Supplementary file 1 [file microorganisms-14-01120-s001.zip › Figure S3.tif]

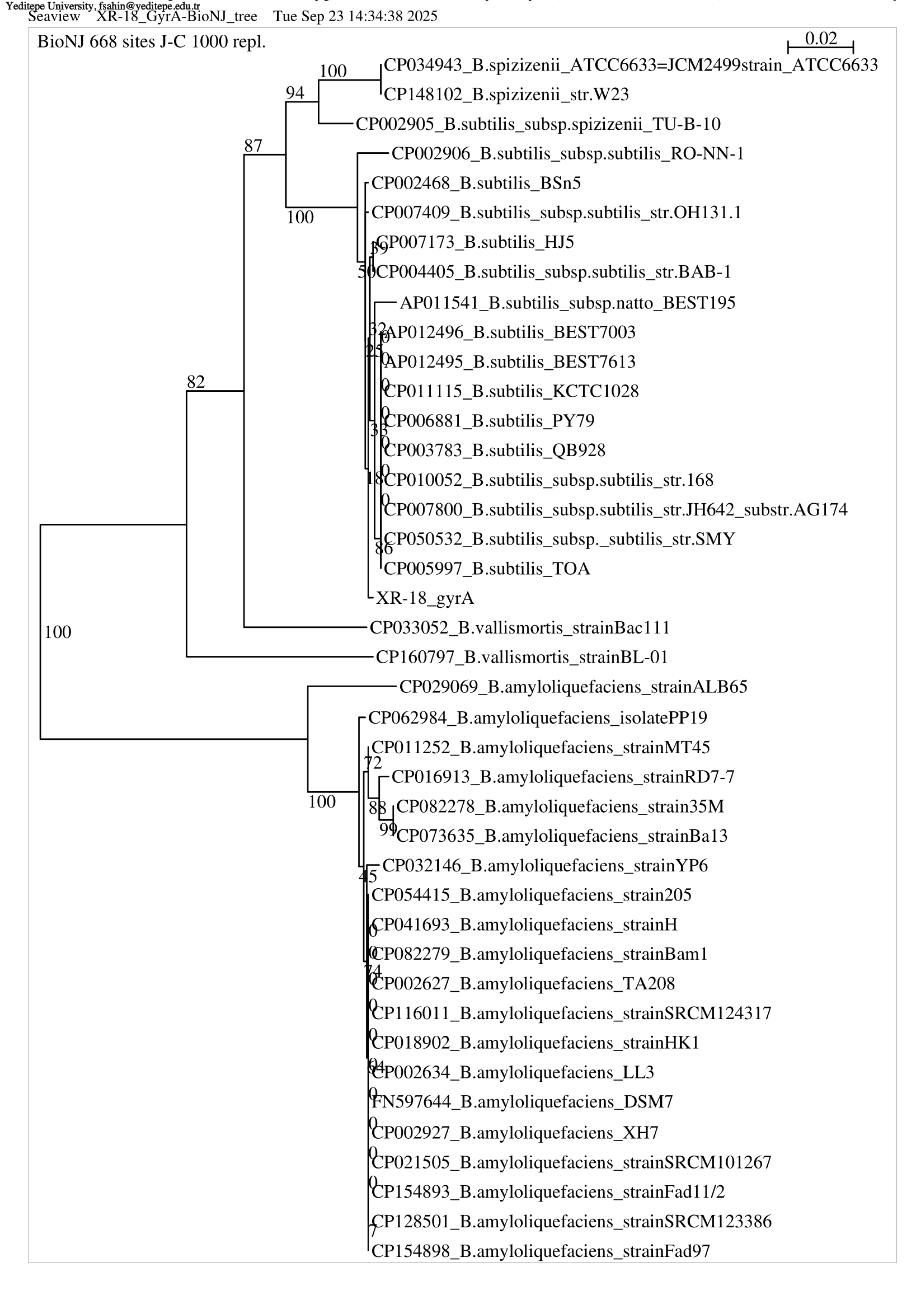

Supplement: Supplementary file 1 [file microorganisms-14-01120-s001.zip › Figure S4.tif]

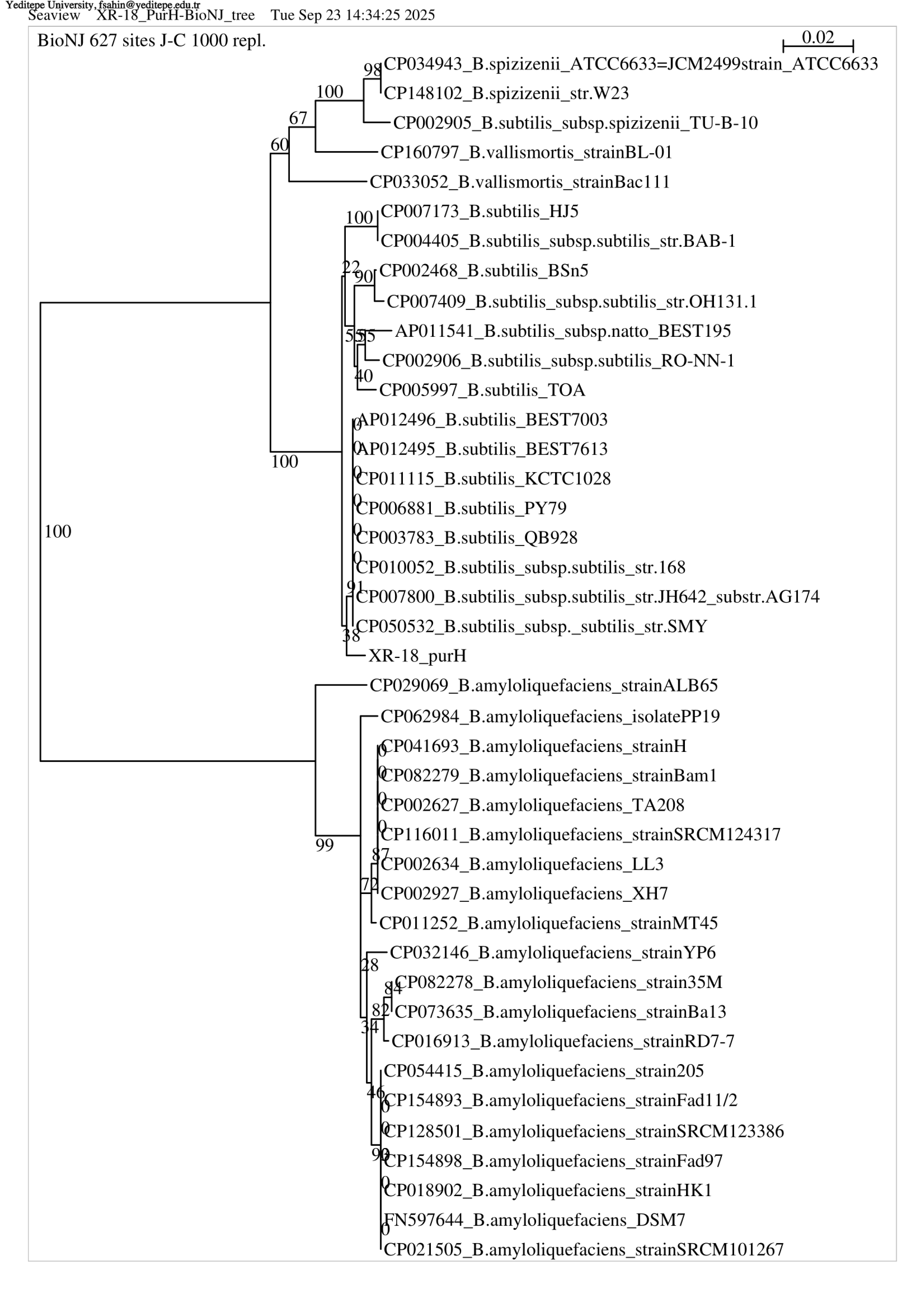

Supplement: Supplementary file 1 [file microorganisms-14-01120-s001.zip › Figure S5.tif]

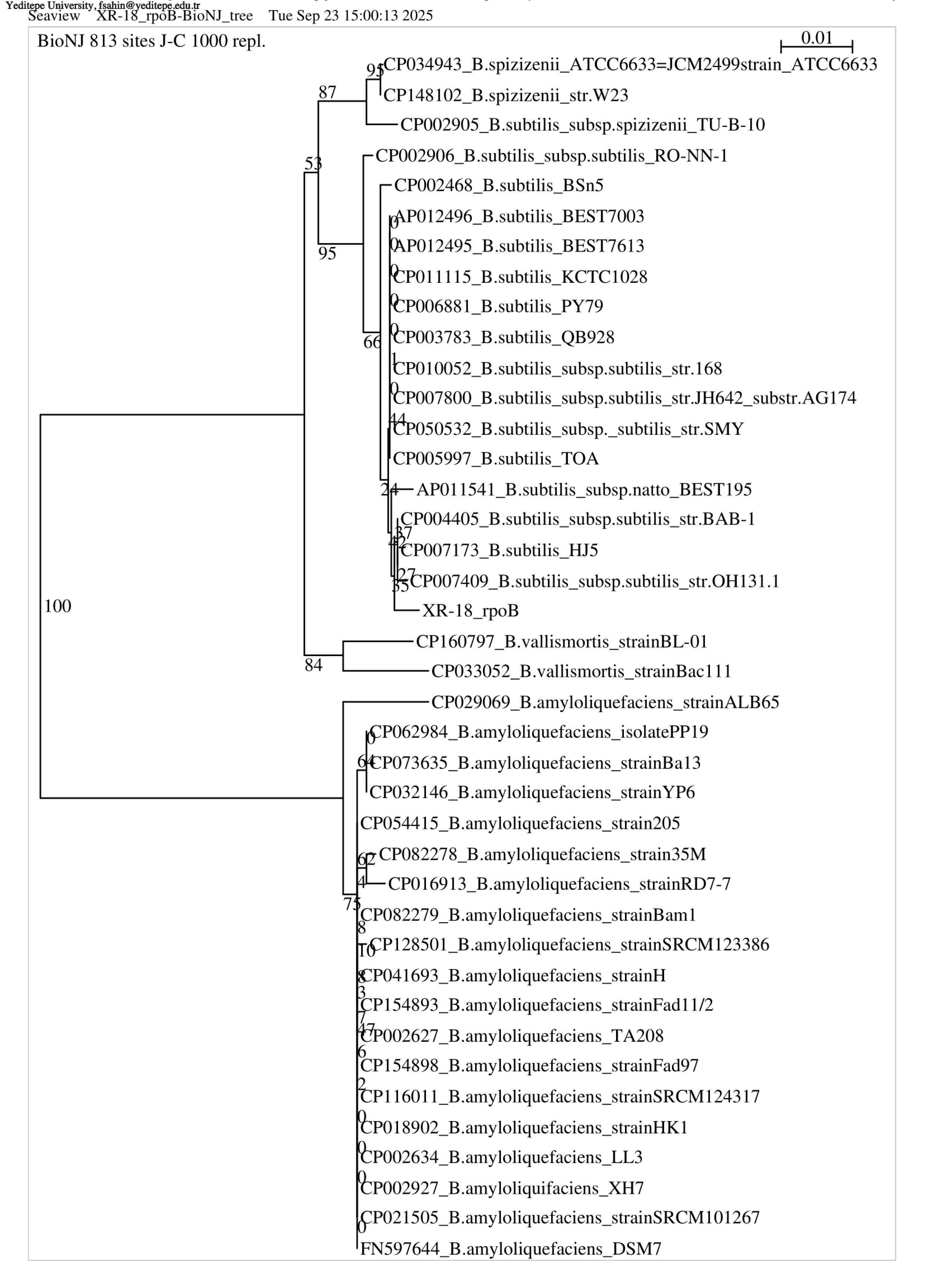

Supplement: Supplementary file 1 [file microorganisms-14-01120-s001.zip › Figure S6.tif]

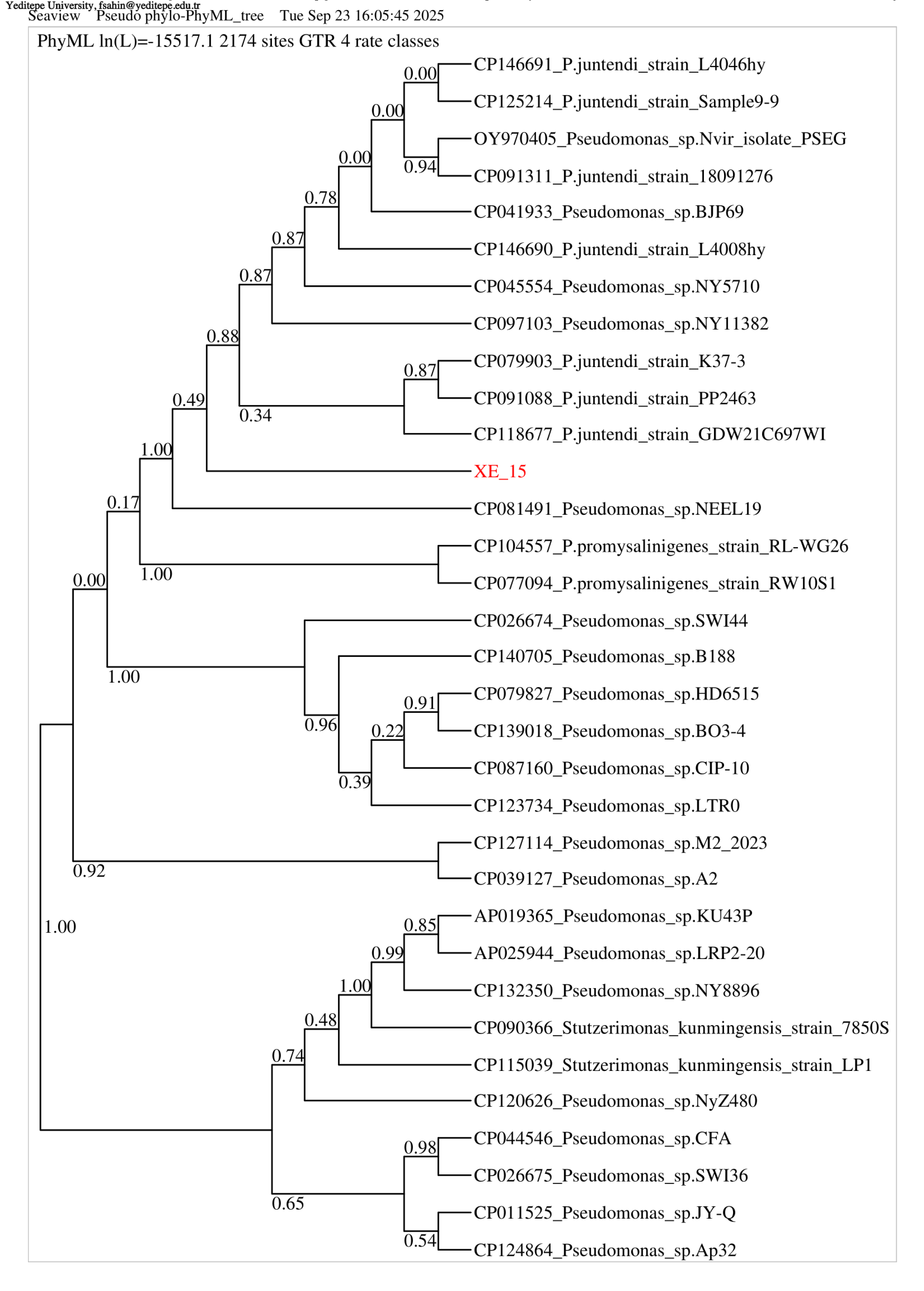

Supplement: Supplementary file 1 [file microorganisms-14-01120-s001.zip › Figure S7.tif]

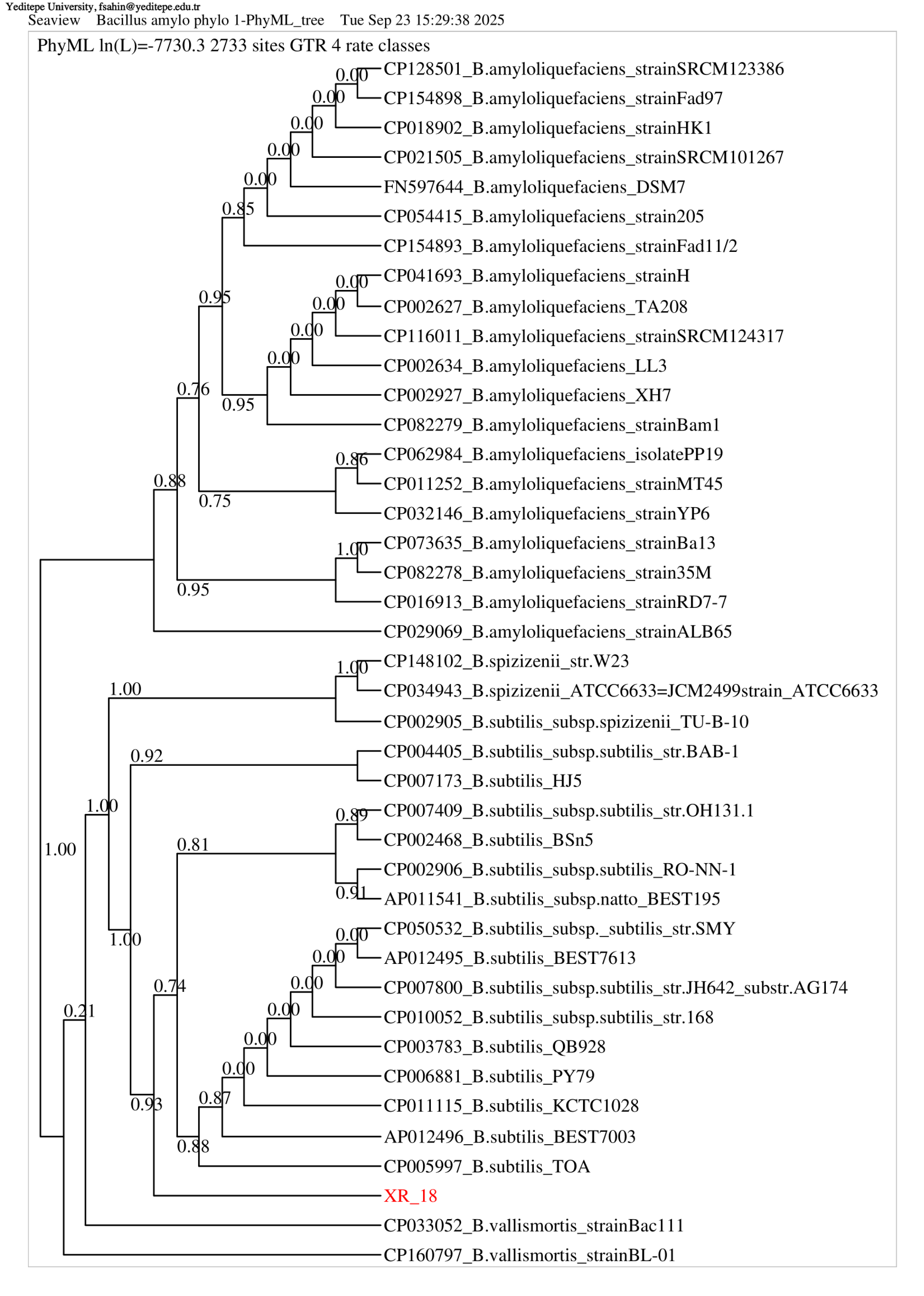

Supplement: Supplementary file 1 [file microorganisms-14-01120-s001.zip › Figure S8.tif]
